# Kinetic Study of the Avocado Sunblotch Viroid Self-Cleavage Reaction Reveals Compensatory Effects between High-Pressure and High-Temperature: Implications for Origins of Life on Earth †

**DOI:** 10.3390/biology10080720

**Published:** 2021-07-28

**Authors:** Hussein Kaddour, Honorine Lucchi, Guy Hervé, Jacques Vergne, Marie-Christine Maurel

**Affiliations:** 1 Department of pharmacology, Renaissance School of Medicine, Stony Brook University, Stony Brook, NY 11794, USA; hussein.kaddour@stonybrook.edu; 2 Société PYMABS, 5 rue Henri Auguste Desbyeres, 91000 Évry-Courcouronnes, France; honorine.lucchi@gmail.com; 3 Laboratoire BIOSIPE, Institut de biologie Paris-Seine, Sorbonne Université, 7 quai Saint-Bernard, 75005 Paris, France; guy.herve@sorbonne-universite.fr; 4 Institut de Systématique, Evolution, Biodiversité, (ISYEB), Sorbonne Université, Museum National d’Histoire Naturelle, CNRS, EPHE, F 75005 Paris, France; jvergne@mnhn.fr

**Keywords:** viroid, hydrostatic pressure, temperature, structure–activity relationship, RNA World

## Abstract

**Simple Summary:**

Viroids remain the smallest infectious agents ever discovered. They are found in plants and consist of single-stranded non-coding circular RNA. Due to their simplicity, viroids are considered relics of an ancient RNA World that may have originated in the deep seas near hydrothermal vents where temperature and pressure are both elevated. To test this hypothesis, a synthetic avocado sunblotch viroid, whose structure contain an autocatalytic hammerhead ribozyme, was subjected to increased pressure (from atmospheric pressure to 300 MPa) at different temperatures (0–65 °C) and the reaction rate constant of the catalytic activity was calculated for each condition. The results obtained allowed calculation of the positive activation volume of this viroid and revealed a compensatory effect between pressure and temperature. In conclusion, these results not only exemplify the plasticity of RNA and support the RNA World hypothesis, but also highlight the usefulness of the hydrostatic pressure in understanding the structure–function relationships of biomacromolecules.

**Abstract:**

A high pressure apparatus allowing one to study enzyme kinetics under pressure was used to study the self-cleavage activity of the avocado sunblotch viroid. The kinetics of this reaction were determined under pressure over a range up to 300 MPa (1–3000 bar). It appears that the initial rate of this reaction decreases when pressure increases, revealing a positive ΔV≠ of activation, which correlates with the domain closure accompanying the reaction and the decrease of the surface of the viroid exposed to the solvent. Although, as expected, temperature increases the rate of the reaction whose energy of activation was determined, it appeared that it does not significantly influence the ΔV≠ of activation and that pressure does not influence the energy of activation. These results provide information about the structural aspects or this self-cleavage reaction, which is involved in the process of maturation of this viroid. The behavior of ASBVd results from the involvement of the hammerhead ribozyme present at its catalytic domain, indeed a structural motif is very widespread in the ancient and current RNA world.

## 1. Introduction

**Figure 1:**
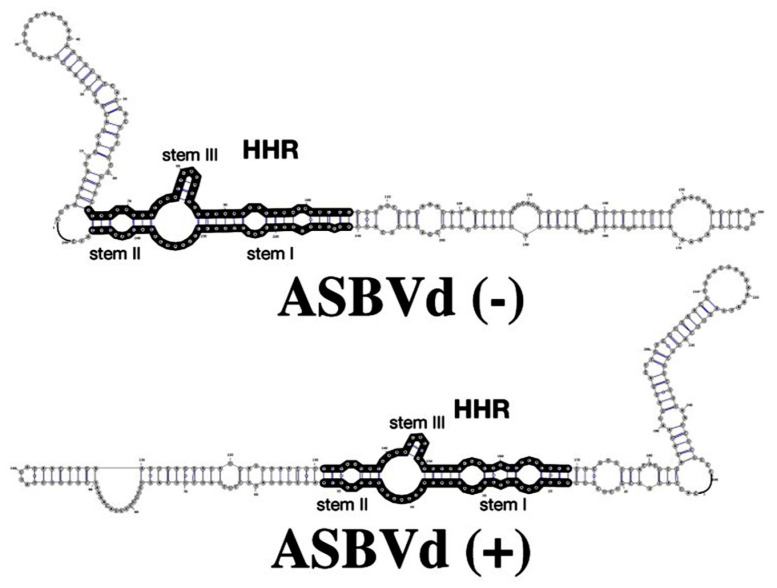
RNA 2D structures of the avocado sunblotch viroid (ABSVd(−)) and ASBVd(+) (top and bottom, respectively). The full-length genome of ASBVd can fold into 2D structures that preserve the hammerhead ribozyme (HHR) motif (regions in black) in both the (−) and (+) strands; the HHR motif of ASBVd(−) is more stable, with 3 base-pairs in stem III but only two base-pairs in ASBVd(+). (Reprinted with permission from ref. [22]. Copyright 2019 Marie-Christine Maurel).

Viroids are the smallest pathogens of plants characterized by a compact rod-like circular RNA 246–401 nucleotides long [1,2]. They have no envelope, no capsid, and they do not code for any protein. Viroids are divided into two families, the Pospiviroidae, and the Avsunviroidae family whose members possess a catalytic RNA with a hammerhead ribozyme (HHR) motif responsible for a crucial cleavage step during viroid replication, such as the avocado sunblotch viroid (ASBVd) [3]. Figure 1 shows the structure of ASBVd with the location of the HHR motif of about 35 nucleotides with a 3D structure composed of three helical junctions (I, II and III) and a core of invariant nucleotides required for its activity.

Chemically, metal ions are involved in HHR activity within the cleavage sites (C–U and C–G) [4]. Cleavage of HHR is a transesterification reaction that converts a 5′, 3′ diester to a 2′, 3′ cyclic phosphate diester via an SN2 mechanism [5]. During replication, (+) and (−) complementary strand sequences of Avsunviroidae are generated through the symmetric rolling circle mechanism. The analysis of the ASBVd contents in avocado extracts [6] revealed the presence of RNA of both polarities in multimeric forms, from monomers to octamers for ASBVd(+) and monomers to dimers for ASBVd(−). This difference in oligomeric sizes reveals a less efficient in vivo cleavage activity of ASBVd(+) than of ASBVd(−) that was observed by in vitro cleavage. The viroid moves within the cell due to intrinsic RNA signals but it is also likely that it recruits supporting protein or RNA factors. Due to the diversity of structures and dynamics that participate in viroid trafficking within the cell and between cells and during infectivity, it is of crucial interest to characterize the structural elements involved in viroid processing. Despite the large amount of information regarding the molecular biology of Avsunviroidae, much less is known regarding the structure and conformational aspects of the cleavage of minus and plus ASBVd strands and the catalytic role of Mg^2+^ in efficient self-cleavage of such viroids.

Over the last few decades, high hydrostatic pressure has been gaining attention as a key thermodynamic and kinetic parameter that brings insights into the structure–activity relationships in biomolecules, such as proteins and nucleic acids, but also as a tool that has been increasingly used in biotechnology [7,8]. Pressure increases the surface of these molecules, which is exposed to the solvent but the volume of their solution decreases. This is due to the electrostriction phenomenon in which water molecules come to pack around the ionized and polar groups presents at the surface of the macromolecule [9]. Pressure favors this process since the negative ΔV of electrostriction is −3 mL/mole of water [10]. Consequently, pressure tends to expose the charged groups to the solvent thus altering the tertiary structure of protein and to dissociate oligomeric proteins leading to the abolition of their biological role and to the inactivation of enzymes. In the case of nucleic acids, this methodology was applied to hairpin [11,12,13] and hammerhead [14] ribozymes, but not yet to complete genomes such as viroids. The previous studies focused only on the minimal self-cleaving sequence of RNA because of the technical difficulties, yet it is likely that sequences outside the catalytic core may affect the global conformational change and thus the fitness of the viroid in vivo. In this regard, Hui Bon Hoa et al. [15,16] studied the structures of both, ASBVd(−) and ASBVd(+) strands (Figure 1), by Raman spectroscopy and showed that both molecules exhibited a typical A-type RNA structure with an ordered double-helical content as expected. Nonetheless, small but specific differences between the two strands were found in the sugar puckering and base-stacking regions. Furthermore, both stands responded differently to deuteration and to the temperature increase, since both conditions differentially perturbed the double-helical content and the phosphodiester conformation of viroids, as revealed by the corresponding Raman spectral changes. These structural differences suggested that the rigidity and stability were higher and the D_2_O accessibility to the H-bonding network was lower for ASBVd(+) compared to ASBVd(−), which correlated to the catalytic activity, ASBVd(−) being 3.5 times more active than ASBVd(+).

Another interesting aspect for the study of viroids’ behavior under high pressures and high temperatures is that viroids are often deemed relics of the “RNA World” [17]. Indeed, viroids are functional nucleic acids that operate under unusual conditions [18] and might have once strived under conditions that are considered today to be extreme. Such conditions are also prevalent in terrestrial subsurface environments, which nurture a massive microbial reservoir, estimated at about 70% of the Earth’s microbial life [19,20]. Some of these bacteria, for instance the subseafloor *Petrocella atlantisensis*, are able to strive in the laboratory, if cultured under high hydrostatic pressure [21]. It is also imaginable that such extreme conditions of high-pressure high-temperature are ordinary on other rocky planets, or even exoplanets. Furthermore the “intraterrestrial” life asked the question: would these intraterrestrials have appeared first? What if life was not born “on” Earth, but “in” Earth? Yet, how exactly viral and subviral particles behave under these conditions is unknown. In this investigation, pressure and temperature and their combination were used to obtain information concerning the activation volume and the activation energy associated to the formation of the transition state of the self-cleavage reaction of the full ASBVd(−) viroid.

## 2. Materials and Methods

### 2.1. In Vitro Synthesis of ASBVd(−)

Experiments were performed on synthetic linearized ASBVd(−) as follows: a previously cloned *pk*S plasmid with the ASBVd monomer between BamH1 and EcoR1 restriction sites [23,24] was subjected to a PCR amplification using 5′-TAATACGACTCACTATAGGAAGAGATTGAAGACGAGTG-3′ and 5′-GATCACTTCGTCTCTTCAGG-3′, as forward and reverse primers, respectively, with the underlined sequence corresponding to the T7 promoter. After a clean-up step by an ethanol precipitation, verification of its size on a 2% agarose gel, and its quantification by ImageJ (NIH, Bethesda, Maryland, USA, https://imagej.nih.gov/ij/, accessed on 13 May 2021), the PCR product was used in an overnight in vitro RNA synthesis at 37 °C as previously detailed [16].

### 2.2. Kinetics Studies

All kinetics reactions, with the exception of one (at 0 °C and atmospheric pressure), were performed using a high-pressure high-temperature apparatus (Top-Industrie, France) as described previously [14]. Briefly, ASBVd(−) was denatured at 94 °C for 1 min and slowly (3 °C/min) renatured until reaching 23 °C, before dilution in the reaction buffer (50 mM HEPES, pH 7.5). The solution was loaded into the apparatus and equilibrated at the given pressure/temperature before starting the reaction by addition of 50 mM MgCl_2_. Aliquots were withdrawn at appropriate times, quenched with stop solution (7 M urea, 0.01% xylene cyanol, and 50 mM EDTA). The reaction products were analyzed by a 10% denaturing PAGE and quantified using ImageJ as previously described [14]. The percentage of cleavage was plotted as a function of time and the plots were fitted to a single-exponential equation: $F=F_{max}(1-e^{-k_{obs}t}$) where *F* is the percentage of the cleaved viroid at time *t*, *F_max_*, the maximum percentage of cleaved viroid, and *k_obs_*, the observed rate constants for cleavage. As the exact significance of the plateau observed is not known, *k_obs_* was not deconvoluted into the rate constants for cleavage (*k_cleav_*) and ligation (*k_lig_*).

### 2.3. ASBVd(−) Structural Modeling

RNAfold WebServer [25,26] (http://rna.tbi.univie.ac.at/cgi-bin/RNAWebSuite/RNAfold.cgi, accessed on 23 May 2021) was used with the default parameters [27] and only the temperature was varied from 0 to 65 °C. To model the ASBVd(−) tertiary structure, the dot-bracket notations of the centroid secondary structures were input in the RNAComposer [28] and the CentroidFold secondary structure prediction method was selected. The pdb structures were visualized using iCn3D, a Web-based 3D Viewer for Sharing 1D/2D/3D Representations of Biomolecular Structures [29]. As for the ASBVd(−) solvent accessibility, it was predicted using RNASol an RNA solvent accessibility prediction WebServer [30].

## 3. Results

### 3.1. Influence of Pressure on the Reaction Rate

**Figure 2:**
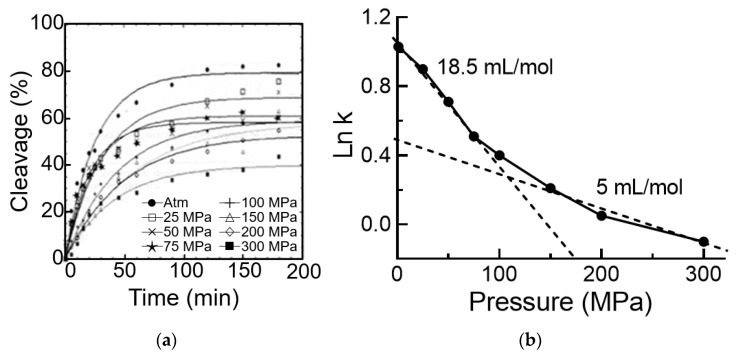
Kinetics of the ASBVd(−) self-cleavage reaction. (**a**) Time course of the reaction at 30 °C and different pressures. (**b**) Plot of Ln k against pressure providing the activation volumes of the reaction.

The self-cleavage activity was measured as indicated in “Materials and Methods” under a range of pressure going from 1 to 300 MPa and at 30 °C. The progress curves obtained are shown in Figure 2a where it can be seen that pressure had a significant negative effect on this reaction, 50% inhibition being observed at 300 MPa. The rate constants of this reaction were used to draw the variation of the Log of the rate constant as a function of pressure. This is shown in Figure 2b. The linear plot of such a representation allows the determination of the activation volume of the reaction, ΔV≠, since the slope of the line is equal to –ΔV≠/RT [31]. In the present case, Figure 2b indicates that at low pressure this activation volume was 18.5 mL/mole and became 5 mL/mole at high pressure. Thus, it appears that pressures inferior to 100 MPa provoke a conformational change in the viroid that is less pronounced at higher pressures (150–300 MPa), although the additional effect was still measurable.

The fact that the slopes were negative indicates that during the formation of the transition state the surface of the viroid exposed to the solvent decreased together with the extent of the electrostriction, due to the exposure of charged and hydrophobic residues to the solvent. This is in accordance with the previous conclusion that the formation of the transition state of this viroid involves a domains’ closure [32]. Thus, pressure had an effect on the initial conformation of the viroid in such a way that its conformation becomes closer to what it was in the transition state of the reaction. The above-described influence of pressure on the activity of the viroid is fully reversible.

### 3.2. Influence of Temperature on the Reaction Rate

**Figure 3:**
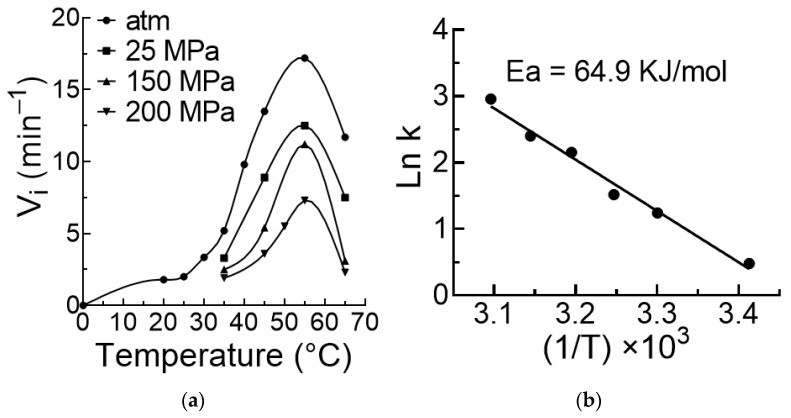
Effects of temperature on the ASBVd(−) self-cleavage reaction. (**a**) Rates of reaction as a function of temperature at different pressures. (**b**) Corresponding Arrhenius plot at atmospheric pressure providing the energy of activation of the reaction.

The temperature dependence of the self-cleavage reaction is shown on Figure 3a. As expected, the temperature increased the rate of the reaction up to 55 °C then it decreased it, irrespective of the pressure. Since the rate of the self-cleavage reaction is independent of the viroid concentration, the initial rates of this reaction directly reflected the rate constants and were thus used here to draw the variation of Ln k as a function of pressure. That did not change the slope of the line obtained and allowed us to graph the Arrhenius plot, which provided a value of 64.9 KJ/mol for Ea, the activation energy of the reaction (Figure 3b).

### 3.3. The 2D and 3D Structural Modeling of ASBVd(−) at Different Temperatures

To gain additional insight into ASBVd(−) conformational changes during autocatalysis, 2D and 3D models depicting the secondary and tertiary structures of ASBVd(−) were simulated at the different experimental temperatures, ranging from 0 to 65 °C. In the 2D models, two prediction methods were used: the minimum free energy prediction and the thermodynamic prediction. The first method yields the “optimal secondary structure”, which has the minimum free energy (MFE), and the second yields the “centroid secondary structure”, which has the minimum total base-pair distance to all structures in the thermodynamic ensemble [27,33]. The MFE for both prediction methods are given in Table 1, showing an expected increase with the temperature.

**Figure 4:**
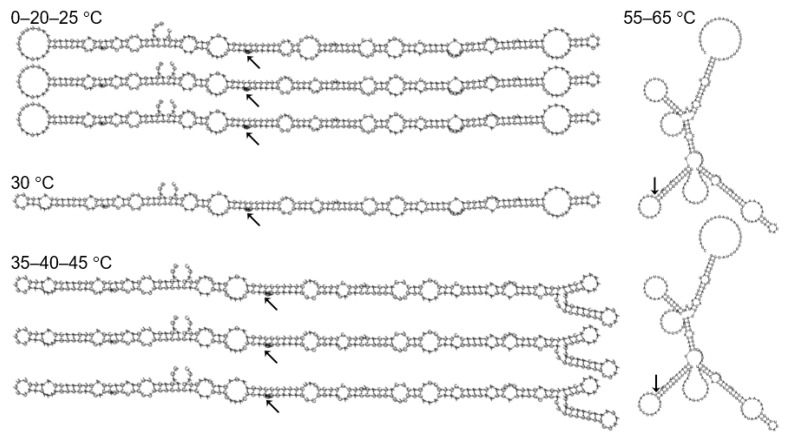
Optimal secondary structures of ASBVd(−) at different temperatures as determined using the RNAfold WebServer. Arrows denote C86, the cleavage site.

The MFE secondary structures, generally rod-like, did not significantly change between 0 and 45 °C, especially around the cleavage site (Figure 4). In fact, structures at 0–20–25 °C were identical, similarly for structures at 35–40–45 °C. Structures at 55 °C and 65 °C, although branched and different from those at lower temperatures, were also identical to each other.

**Figure 5:**
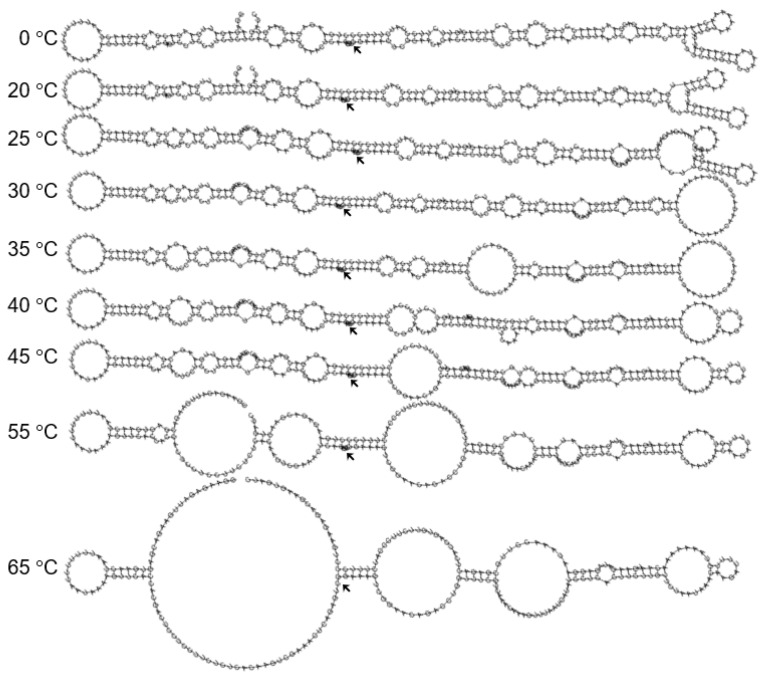
Centroid secondary structures of ASBVd(−) at different temperatures as determined using the RNAfold WebServer. Arrows denote C86, the cleavage site.

**Figure 6:**
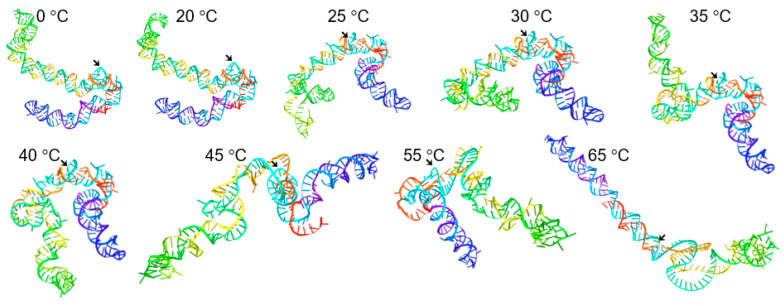
The 3D structures of ASBVd(−) at different temperatures as determined using the RNAComposer. The dot-bracket notations of the centroid secondary structures shown in Figure 5 were used as input and the CentroidFold secondary structure prediction method was selected. Arrows denote C86, the cleavage site. The nucleotides are rainbow-colored (3′-red, orange, yellow, green, turquoise, blue, indigo, and violet-5′). 3′ and 5′ indicate the 3′-OH and 5′-phosphate extremities of the viroid.

**Figure 7:**
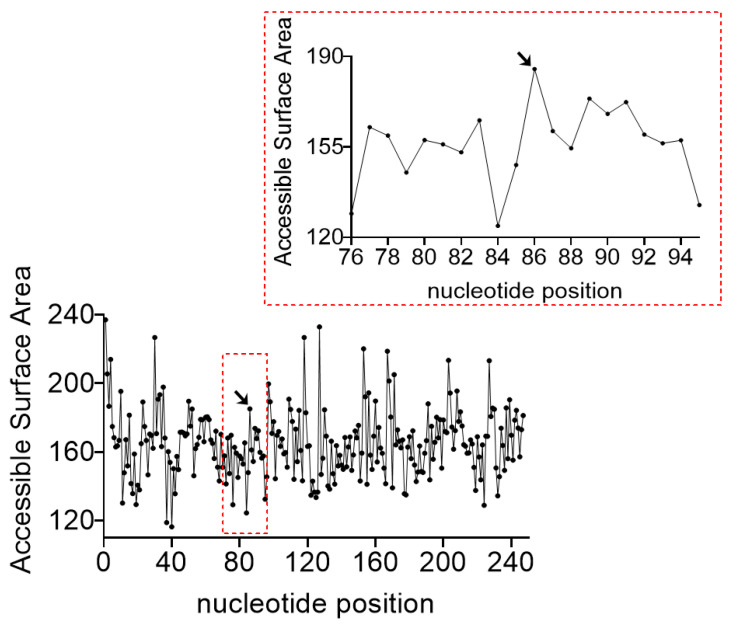
Accessible surface area of ASBVd(−) nucleotides as determined using the RNASol WebServer. The inset is the enlarged graph around the cleavage site, denoted with an arrow.

On the other hand, the centroid secondary structures were unique at each temperature and showed an expected gradual opening of the molecule, although the overall rod-like structure was persistent (Figure 5). Furthermore, the 3D tertiary structure, calculated using these centroid secondary structures as input, were also different at different temperatures (Figure 6). Specifically, at 0–30 °C the structures were largely twisted, at 35 °C both ends were antiparallel, at 40 °C a molecular closure in which the two ends came close to each occurred, and this was more pronounced at 55 °C. Interestingly at 45 °C, the viroid assumed a unique ohm-shape conformation in which the cleavage site pointed to the inside of the structure, an indication of a possible change in the solvent accessibility, and a novel active conformation for catalytic RNAs. Finally at 65 °C, the viroid assumed a partially open and less twisted structure, although the rod-like structure around the cleavage site was still maintained. In addition, ASBVd(−) accessibility to the solvent was also predicted and the result showed that, unsurprisingly, the cleavage site had the highest solvent accessible surface area among its surrounding nucleotides (Figure 7). Altogether, these results illustrated the importance of the conformational changes that accompany the RNA autocatalysis.

### 3.4. Reciprocal Effects of Pressure and Temperature on the Activation Energy

**Figure 8:**
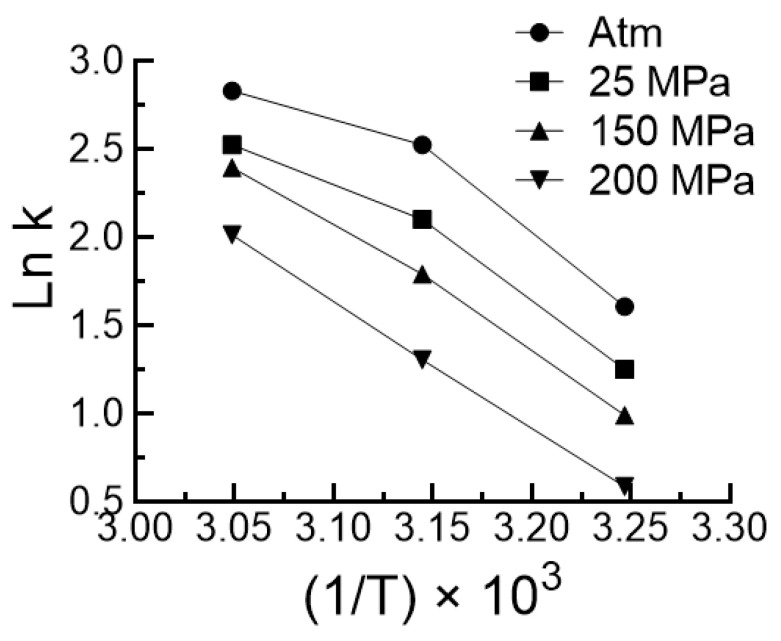
Arrhenius plots of the autocleavage reaction of ASBVd(−) at different pressures.

Pressure had no influence on the temperature dependence of the self-cleavage reaction as explained below. This temperature dependence was determined under pressures going from 1 to 200 MPa. As shown in Figure 3a the temperature dependence profiles were exactly the same at all pressures. The rate of reaction was measured at four pressures and three temperatures. The results are shown in Figure 8. Although accurate energy of activation cannot be calculated on three points, the interesting observation is that the plots of Ln k f(1/T) obtained at various pressures were nearly parallels, indicating that the energy of activation of the reaction was not significantly affected by pressure.

**Figure 9:**
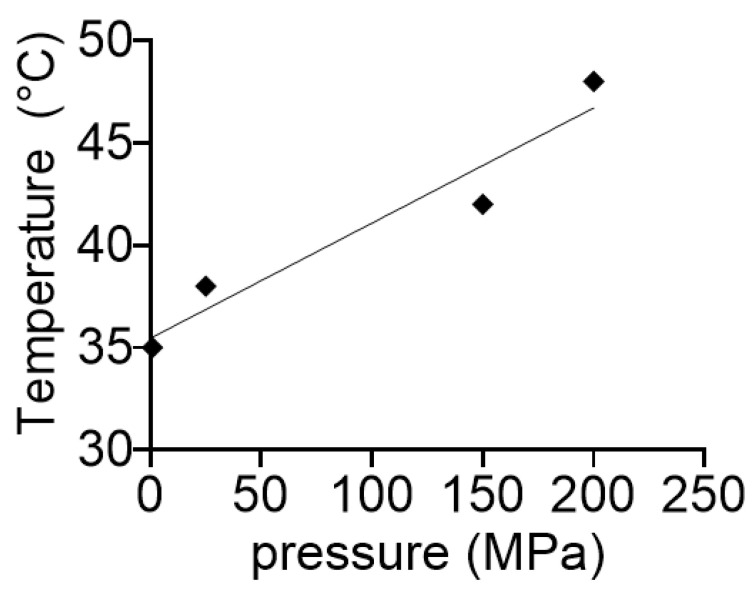
Reciprocal compensatory effects of pressure and temperature in the ASBVd(−) autocatalytic reaction. The plot was determined by estimating the temperature required at each pressure to achieve a 5% cleavage of the viroid in 1 min.

Pressure and temperature exert reciprocal compensatory effects. This is visualized in Figure 9. This property illustrates the adaptability of RNA molecules and viroids in particular. It might have played an important role in the early development of life, particularly in the environment of the deep-sea vents, which have been proposed to be the cradle of life [34].

## 4. Discussion

The importance of hydrostatic pressure and temperature as critical physical parameters in the understanding of the structure–function relationships of enzymes has long been established [35,36,37,38,39]. Regarding small autocatalytic RNAs, or ribozymes, the conformational changes accompanying the catalytic activity have also been characterized. For instance, a hairpin ribozyme requires a ΔV≠ ranging between 23 and 34 mL/mole depending on the particular RNA [11,12,13]. The hairpin ribozyme ΔV≠ was about 2–3 fold larger than that of a viroid-derived hammerhead ribozyme, ranging between 3 and 12 mL/mole and reflecting the presence in the solution of two isomer populations with different conformations [14]. In this study, ASBVd(−) also exhibited at least two conformers with measurable ΔV≠ of 5 and 18.5 mL/mole (Figure 2b). The fact that ΔV≠ of the entire ASBVd(−) was similar to that of the minimal ribozymic motif suggests that the measured activation volume corresponds exclusively to a local conformational change near the catalytic site and the viroid region outside the catalytic core does not play a significant role in the catalytic reaction. This explanation is in-line with the hypothesis that the viroid sequences outside the catalytic core have essentially evolved to stabilize its overall structure and optimize its activity.

Another interesting observation is that a relatively slow ASBVd(−) autocatalytic reaction had a ΔV≠ comparable to that of a fast-cleaving minimal hammerhead ribozyme, indicating that ΔV≠ alone does not necessarily correlate with enzymatic activity, and rather depends on the structural moieties of the molecule. For instance, the lower activity of ASBVd(−) compared to the minimal hammerhead and hairpin ribozymes may be due to the relatively larger size of the viroid requiring more Mg^2+^ ions to shield the phosphate groups, before triggering the specific chemical reaction of cleavage. Furthermore, it is possible that large regions outside the catalytic site might be at play, in such a way that despite the large conformational changes, the overall ΔV≠ is compensated and, hence, small. In this regard, ASBVd(−) tertiary structure modeling at different temperatures, revealed that, indeed, both ends of the rod-like structure of the viroid were involved in large conformational changes in a way that the structure bent around the cleavage site at 55 °C, where the viroid was most active. This molecular closure was similar to that of the small ribozymes.

The temperature-dependent activity is extensively studied in the case of small ribozymes [40,41,42,43,44]. However, little is known about the temperature dependence of viroids self-cleavage activity. It is shown here that, as expected, ASBVd(−) autocatalytic activity increased with temperature up to 55 °C and then rapidly dropped above 55 °C, and that also occurred at different pressures (Figure 3a). With this strong dependency on temperature, one could expect that the energy of activation would change under pressure. For instance, the higher the pressure, the slower the catalytic reaction will be, and, as a consequence, the higher the activation energy. However, this was not the case (Figure 8). A possible explanation could be that the domain closure contributes poorly to the energy of activation, which could be essentially linked to the chemistry of the reaction. By contrast, pressure and temperature have had compensatory effects on the autocatalytic reaction (Figure 9). This finding has direct relevance to the possible sites of the origins of life on Earth, where viroids may have evolved [45], and suggests that near hydrothermal vents environments, where pressure and temperature are high [46], may elicit a comparable activity to that occurring at warm ponds on the early Earth’s surface [47]. The significance of these results also extend to the extant biology where ribozymes are omnipresent and ultraconserved, particularly in the human genome [48].

## 5. Conclusions

In summary, the present study reported measurable compensatory effects between pressure and temperature on the self-cleavage activity of ASBVd(−). We conclude that viroids, despite their simplicity, exhibit exceptional plasticity and adaptability to a wide array of physical conditions that may have once existed on early Earth.

## Figures and Tables

**Table 1:** Minimum free energy (MFE) ^1^ of the different secondary structures of ASBVd(−) at different temperatures.

Temperature (°C)	Minimum Free Energy Prediction (kcal/mol)	Thermodynamic Ensemble Prediction (kcal/mol)
0	−118.4	−119.71
20	−83.78	−86.79
25	−75.16	−78.84
30	−66.63	−71.05
35	−58.54	−63.44
40	−50.75	−56.11
45	−43.13	−49.09
55	−28.64	−36.01
65	−17.16	−24.64

^1^ Values determined using the RNAfold WebServer with the default parameters.

## Data Availability

The data presented herein are available from the corresponding author upon a reasonable request.
